# External electric field effects on the σ-hole and lone-pair hole interactions of group V elements: a comparative investigation†

**DOI:** 10.1039/d0ra09765a

**Published:** 2021-01-19

**Authors:** Mahmoud A. A. Ibrahim, Sherif M. A. Saad, Jabir H. Al-Fahemi, Gamal A. H. Mekhemer, Saleh A. Ahmed, Ahmed M. Shawky, Nayra A. M. Moussa

**Affiliations:** Computational Chemistry Laboratory, Chemistry Department, Faculty of Science, Minia University Minia 61519 Egypt m.ibrahim@compchem.net; Chemistry Department, Faculty of Applied Sciences, Umm Al-Qura University Makkah 21955 Saudi Arabia; Chemistry Department, Faculty of Science, Assiut University Assiut 71516 Egypt; Science and Technology Unit (STU), Umm Al-Qura University Makkah 21955 Saudi Arabia; Central Laboratory for Micro-analysis, Minia University Minia 61519 Egypt

## Abstract

σ-hole and lone-pair (lp) hole interactions of trivalent pnicogen-bearing (ZF_3_) compounds were comparatively scrutinized, for the first time, under field-free and external electric field (EEF) conditions. Conspicuously, the sizes of the σ-hole and lp-hole were increased by applying an EEF along the positive direction, while the sizes of both holes decreased through the reverse EEF direction. The MP2 energetic calculations of ZF_3_⋯FH/NCH complexes revealed that σ-holes exhibited more impressive interaction energies compared to the lp-holes. Remarkably, the strengths of σ-hole and lp-hole interactions evolved with the increment of the positive value of the considered EEF; *i.e.*, the interaction energy increased as the utilized EEF value increased. Unexpectedly, under field-free conditions, nitrogen-bearing complexes showed superior strength for their lp-hole interactions than phosphorus-bearing complexes. However, the reverse picture was exhibited for the interaction energies of nitrogen- and phosphorus-bearing complexes interacting within lp-holes by applying the high values of a positively directed EEF. These results significantly demonstrate the crucial influence of EEF on the strength of σ-hole and lp-hole interactions, which in turn leads to an omnipresent enhancement for variable fields, including biological simulations and material science.

## Abstract 2

σ-hole and lone-pair (lp) hole interactions of trivalent pnicogen-bearing compounds were comparatively investigated under field-free and external electric field (EEF) conditions.

## Graphical abstract

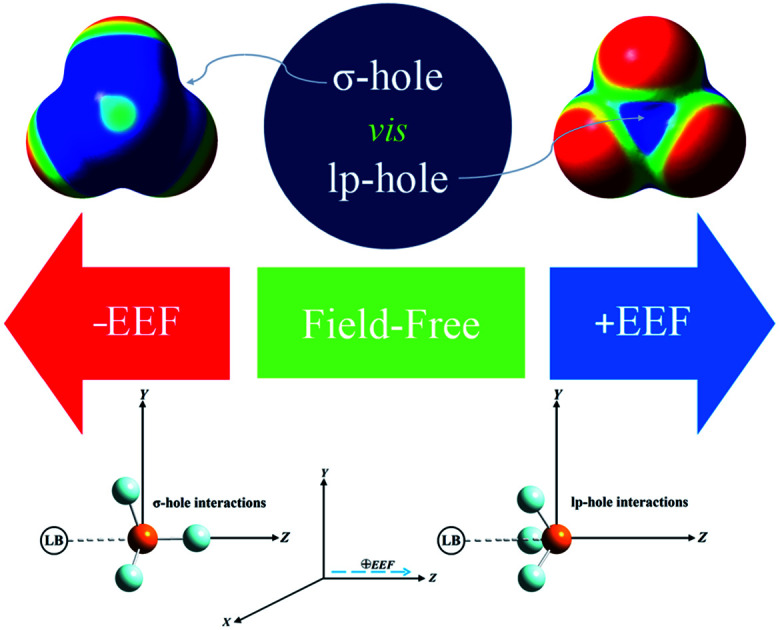

## Introduction

In recent years, veritable growing attention has been directed towards the conceptualization and characterization of all categories of noncovalent interactions, a phenomenon that can reasonably be ascribed to their important roles in chemistry^1,2^ and biochemistry.^3–5^ In addition to the traditional investigation of noncovalent interactions, great attention has been paid to studying σ-hole interactions.^6–10^ Based on the chemical family to which the Lewis acid central atom belongs, for groups IV–VII in the periodic table, σ-hole interactions have been labeled as tetrel,^11–13^ pnicogen,^14–16^ chalcogen,^17–21^ and halogen^22,23^ bonds, respectively. Among those interactions, pnicogen bonding plays a vital role in supramolecular chemistry^24,25^ and crystal engineering.^26^ Along with σ-holes, which lie along the extension of the covalent bonds, pnicogen-bearing molecules can also develop lp-holes, and appear directly opposite to lone pairs and interact with Lewis bases to form lp-hole-bonded complexes.^27–30^

As a point of departure, the intensive local electric field arising from the medium surrounding biological systems significantly affects noncovalent interactions existing in biomolecules.^31,32^ Recent studies confirmed the crucial importance of the external electric field (EEF) as a potent effector for future smart and green reagents.^33–35^ As a matter of fact, the electric field was found to have an undisputed impact on catalysis, bond dissociation, regioselectivity, stereoselectivity, mechanistic crossover, and inhibition.^36–41^ Thus far, it has been found that the variability of the electric field effects on the reactivity of reactions is essentially relevant to the microscopic field orientation.^34^ Additionally, EEF can potentially be employed to deploy unprecedented control over chemical reactivity, in turn leading to the implementation of versatile and unconventional synthetic tools in organic and biochemistry fields.^32,42–44^

Very recently, various studies were carried out to resolve and identify the contribution of the external electric field (EEF) in regulating the nature and strength of noncovalent interactions.^32,45–51^ Numerous intriguing studies highlighted the vital influence of EEF on the basic features of halogen-based interactions.^52,53^ In this spirit, the employed EEF could potentially be utilized to tune a traditional Cl⋯N halogen bond to a chlorine-shared or an ion-pair bond. The EEF direction also has a remarkable effect on the strength of group VII interactions. Through applying EEF along the *z*-axis in the positive direction, an impressive enhancement of the strength of halogen-based interactions was obviously obtained. Apparently, cation⋯π interactions between benzene and alkali metal ions were exposed to EEF, which theoretically demonstrated the dependence of the interaction strength on the magnitude and direction of the applied EEF.^50^ In line with cation⋯π interactions, anion-containing candidates were proclaimed to have significant sensitivity to the influence of EEF.^51^ Moreover, the effects of EEF on π–π stacking, hydrogen bonding, and X–H⋯π interactions were documented.^49^

A detailed study was herein initiated to compare σ-hole and lp-hole interactions in pnicogen-bearing complexes (*i.e.*, ZF_3_⋯FH/NCH, where Z = N and P) and assess the EEF effect on these interactions. Geometrical optimization, molecular electrostatic potential (MEP), and maximum positive electrostatic potential (*V*_s,max_) calculations were performed on investigated pnicogen-bearing monomers under field-free and directed EEF conditions. Toward a profound insight, the energetic study of optimized pnicogen-bearing complexes was addressed using MP2 and CCSD/CBS levels of calculations. The quantum theory of atoms in molecules (QTAIM) and the noncovalent interaction (NCI) index were established to clarify the effects of EEF on the nature of inspected complexes. σ-hole and lp-hole electrostatic interactions were also explored with the incorporation of the point-of-charge (PoC) approach. PoC results were validated on ZF_3_⋯NCX (where X = F, Cl, Br, and I). The results of this study provide systemic manifestations for future research related to the two main categories of noncovalent interactions, which give rise to sizeable contributions to material science and crystal engineering fields.

## Computational methods

**Fig. 1:**
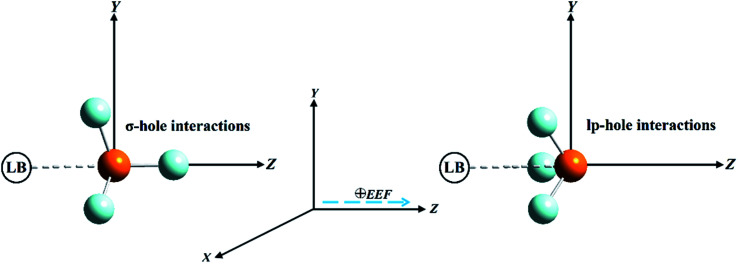
Illustrative representation of the directed external electric field (EEF) imposed on σ-hole⋯ and lp-hole⋯Lewis base (LB) interactions of pnicogen-bearing complexes.

Pnicogen-bearing monomers ZF_3_ with Z = N and P and the binary ZF_3_⋯FH/NCH complexes were optimized under field-free conditions and the influence of an external electric field (EEF) by the second-order Møller–Plesset perturbation theory (MP2) method^54^ with the aug-cc-pVTZ basis set.^55–57^ The employed EEF was oriented along the *z*-axis in both the positive and negative directions, with values ranging from 0.002 to 0.032 au (Fig. 1). Vibrational frequency calculations were not performed for the binary complexes; thus, there was a possibility that the structures were not energetic minima. As a preliminary study, the electrostatic potential analysis was accomplished for the considered monomers to visualize the σ-hole and lp-hole sizes and evaluate their numerical values. In turn, MEP maps and maximum positive electrostatic potential (*V*_s,max_) values were generated using a 0.002 au electron density envelope based on literature recommendations.^58,59^

Interaction energies were calculated for the optimized complexes as the difference in energy between the complex and the sum of the monomers (with the same geometries they adopt within the complex) at the MP2/aug-cc-pVTZ level of theory. Interaction energies were also benchmarked at the CCSD(T)/CBS level for purposes of comparison and validation, as illustrated in eqn (1):^60^

1 *E*_CCSD(T)/CBS_ = Δ*E*_MP2/CBS_ + Δ*E*_CCSD(T)_

where:

2 Δ*E*_MP2/CBS_ = (64*E*_MP2/aug-cc-pVQZ_ − 27*E*_MP2/aug-cc-pVTZ_)/37

3 Δ*E*_CCSD(T)_ = *E*_CCSD(T)/aug-cc-pVdZ_ − *E*_MP2/aug-cc-pVDZ_

Both the MP2 and CCSD(T) energetic quantities were corrected for basis set superposition error (BSSE) by incorporating the counterpoise procedure (CP).^61^ To provide genuine insight into the nature of the investigated complexes, a plethora of topological parameters were elucidated by incorporating quantum theory of atoms in molecules (QTAIM).^62^ In this context, bond critical points (BCPs) and bond paths (BPs) were generated; also, the electron density (*ρ*_b_), Laplacian (∇^2^*ρ*_b_), and total energy density (*H*_b_) were calculated. Furthermore, the noncovalent interaction (NCI) index was applied to further understand the origin of the pnicogen bonds in the complexes under study based on electron density and its derivatives.^63^

Moreover, the Lewis basicity contributions to the strengths of the σ-hole and lp-hole interactions were electrostatically elucidated for the considered pnicogen-bearing complexes using the point-of-charge (PoC) approach.^30^ In the PoC calculations, molecular stabilization energies for the optimized monomers were computed under the EEF influence and the field-free conditions in the presence of −0.25, −0.50, −0.75, and −1.00 au PoCs at an N/P⋯PoC distance ranging from 2.5 to 6.0 Å with a step size of 0.1 Å. The molecular stabilization energies were computed as follows:^64–67^

4 *E*_stabilization_ = *E*_pnicogen-containing molecule-PoC_ − *E*_pnicogen-containing molecule_

Toward an in-depth investigation of the Lewis basicity role in pnicogen-based interactions, the NF_3_⋯ and PF_3_⋯NCX complexes (where X = F, Cl, Br, and I) were fully optimized at the MP2/aug-cc-pVTZ level of theory under field-free and directed EEF conditions. The basis set of aug-cc-pVTZ-PP was used for the heavy Br and I atoms to treat the relativistic effects.^68^ Based on the latter optimized complexes, energetic calculations were also performed at the same geometrical optimization level.

*V*_s,max_ calculations, QTAIM, and NCI index analyses were performed using Multiwfn 3.7 software^69^ and visualized with Visual Molecular Dynamics (VMD) software.^70^ All remaining calculations that did not require external software were carried out using Gaussian 09 software.^71^

## Results and discussion

### Electrostatic potential analysis

**Fig. 2:**
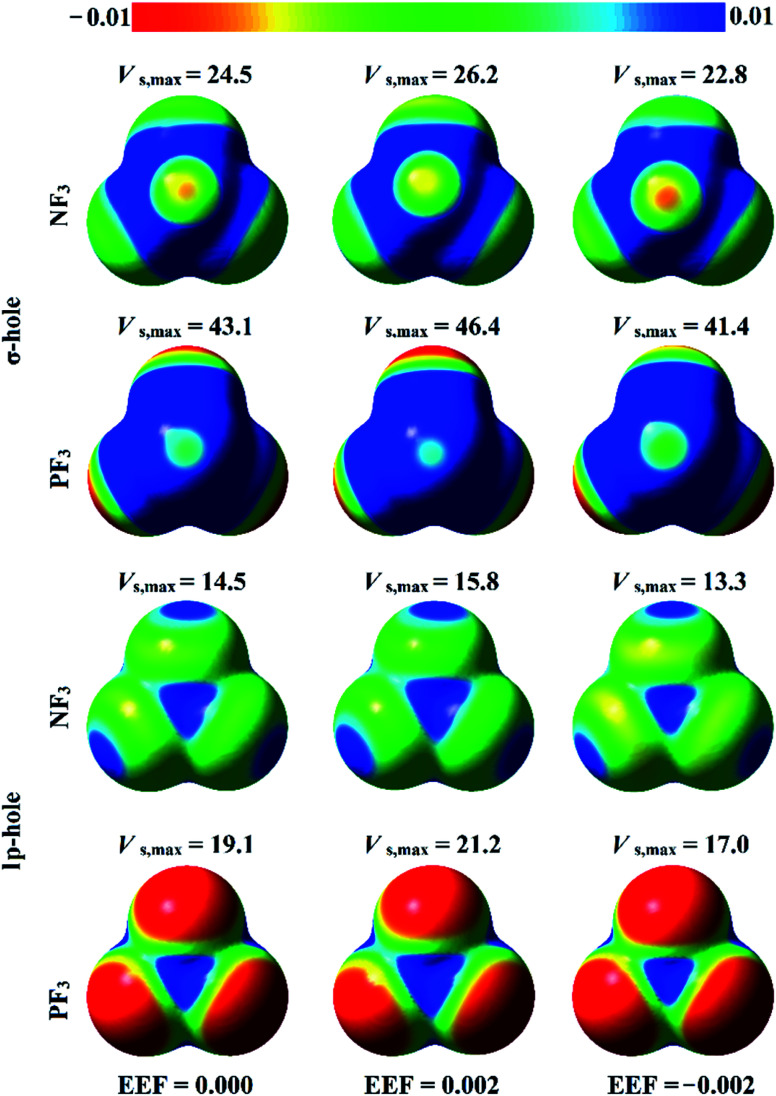
Molecular electrostatic potential (MEP) maps plotted onto 0.002 au electron density contours for NF_3_ and PF_3_ molecules under 0.000 (*i.e.*, field-free), +0.002, and −0.002 au external electric fields (EEFs). The electrostatic potential varies from −0.01 au (red) to +0.01 au (blue). The maximum positive electrostatic potentials (*V*_s,max_) at the σ-hole and lp-hole are computed in kcal mol^−1^.

The analysis of electrostatic potential (EP) is an informative tool for identifying the nucleophilic and electrophilic sites on the molecular surfaces of chemical systems.^72^ Consequently, EP analysis has been employed in numerous studies to explore the potentiality of σ-hole-containing molecules to engage in inter- and intra-molecular interactions.^73–75^ In the current study, EP analysis was performed to demonstrate the electron-deficient and electron-rich sites on the molecular surfaces of pnicogen-bearing molecules. Molecular electrostatic potential (MEP) maps were generated for the optimized ZF_3_ monomers using an 0.002 au electron density contour with EEF values ranging from 0.000 to 0.032 au that aligned along the *z*-axis in the positive and negative directions (Fig. S1 and S2,† respectively). Fig. 2 illustrates MEP maps of NF_3_ and PF_3_ molecules under 0.000 (*i.e.*, field-free), +0.002, and −0.002 EEF conditions as an example.

-As shown in Fig. 2, the occurrence of σ-holes and lp-holes on the surfaces of the considered pnicogen-bearing molecules was demonstrated. Inspecting the sizes of the pictorial holes revealed the favorabilities of the pnicogens to interact *via* σ-holes rather than lp-holes with Lewis bases. Through employing the EEF, the sizes of the σ-holes and lp-holes were increased by directing the utilized EEF in the positive direction, whereas both of them were decreased by applying the EEF in the reverse direction (*i.e.*, the negative direction). Generally, the PF_3_ molecule exhibited a more prominent lp-hole than the NF_3_ analogs. Surprisingly, the lp-hole of N pronounced a larger positive region size than P in the ZF_3_ systems under the influence of a high negatively directed EEF value. Taken together, these results confirm the importance of the EEF directionality and strength in the nucleophilic and electrophilic character of the lp-hole-bearing molecules (Fig. S2†).

**Fig. 3:**
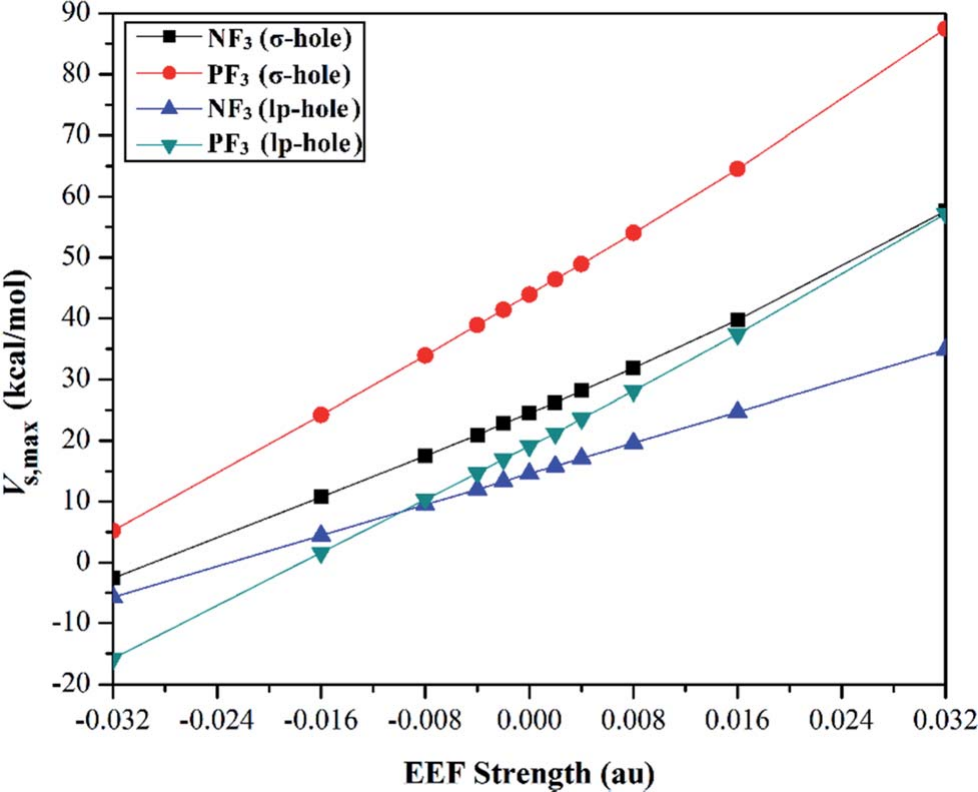
Correlation between the external electric field (EEF) strength and the maximum positive electrostatic potential (*V*_s,max_) value. The positive and negative charges of the EEF were utilized to express the positive and negative directions, respectively.

Quantification of the σ-hole and lp-hole was performed by estimating the maximum positive electrostatic potential (*V*_s,max_) values for all optimized monomers (Fig. 2, S1 and S2†). The correlations between the EEF strength and direction and the *V*_s,max_ value at the σ-hole and lp-hole in the examined pnicogen-bearing molecules are given in Fig. 3.

Looking at Fig. S2,† it can be noted that the *V*_s,max_ value increased with increasing atomic size of the pnicogen atom in the considered molecules (*i.e.*, NF_3_ < PF_3_), with the exception of the values generated for the lp-hole under the influence of the high EEF strength in the negative direction (*i.e.*, the −0.016 and −0.032 au EEFs). In line with the MEP maps, the numerical values of *V*_s,max_ for the σ-hole and lp-hole of all the considered pnicogen-bearing molecules were found to increase and decrease by applying the EEF in the positive and negative directions, respectively (Fig. 3). For instance, the σ-hole of the NF_3_ molecule exhibited *V*_s,max_ values of 28.1, 21.0, and 24.5 kcal mol^−1^ under the influence of +0.004, −0.004, and 0.000 au EEFs, respectively. In all instances, the σ-hole showed larger *V*_s,max_ values than the lp-hole, with values of 24.5 and 43.1 kcal mol^−1^ for NF_3_ and PF_3_, respectively, under the field-free conditions as a case study. Ultimately, discernible enhancements in the predilection of the examined pnicogen-bearing molecules to interact as Lewis acid centers were fulfilled by applying EEF in the positive direction. In contrast, the negatively directed EEF restricted the potentiality of the systems described above to interact with Lewis bases favorably. These results are highly consistent with literature related to the effects of EEFs on noncovalent interactions.^52,53^ Based on these observations, in the forthcoming sections, calculations were performed for the investigated complexes under the field-free conditions and under the influence of the EEF in the positive direction only.

### Energetic study

**Fig. 4:**
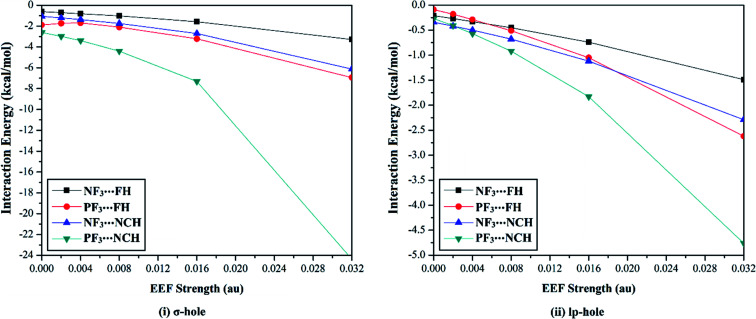
Interaction energies of the ZF_3_⋯FH/NCH complexes (where Z = N and P) calculated for (i) σ-hole and (ii) lp-hole interactions under the influence of a positively directed external electric field (EEF) and under field-free conditions.

The versatility of NF_3_ and PF_3_ molecules to interact with FH and NCH molecules as Lewis bases at σ-hole and lp-hole extensions was comparatively demonstrated, for the first time, under the influence of EEF and field-free conditions. When each pnicogen-bearing molecule was subjected to interaction with the Lewis bases, two geometrical structures for the NF_3_⋯ and PF_3_⋯LB complexes were identified and exemplified based on the interacting hole (*i.e.*, the σ-hole or lp-hole). First, the geometrical structures of the studied complexes were fully optimized at the MP2/aug-cc-pVTZ level of theory under the positively directed EEF, with values ranging from 0.002 to 0.032 au. Second, the interaction energies were computed for the optimized complexes at the same level of theory as the geometry optimization and then benchmarked at CCSD/CBS(T). Fig. 4 illustrates the correlations between the strength of the employed EEF and the interaction energies of the investigated σ-hole⋯ and lp-hole⋯FH/NCH complexes. The results of the energetic study are set out in Table 1.

**Table 1:** Interaction energies calculated (in kcal mol^−1^) at the MP2/aug-cc-pVTZ (*E*_1_) and CCSD(T)/CBS (*E*_2_) levels of theory for the ZF_3_⋯Lewis base optimized complexes under the influence of a positively directed external electric field (EEF) and under field-free conditions (*i.e*., EEF = 0.000 au)

Complexes		EEF (au)	ZF_3_⋯FH				ZF_3_⋯NCH			
			Distance (Å)	Angle^a^ (*θ*)	*E*_1_ (kcal mol^−1^)	*E*_2_ (kcal mol^−1^)	Distance (Å)	Angle^a^ (*θ*)	*E*_1_ (kcal mol^−1^)	*E*_2_ (kcal mol^−1^)
σ-hole	NF_3_⋯LB	0.000	2.97	173.4°	−0.61	−0.75	3.12	175.6°	−1.06	−1.09
		0.002	3.02	175.3°	−0.71	−0.87	3.11	177.1°	−1.21	−1.24
		0.004	2.94	172.6°	−0.81	−0.98	3.10	177.2°	−1.38	−1.41
		0.008	2.91	178.1°	−1.02	−1.20	3.07	177.8°	−1.75	−1.80
		0.016	2.85	177.4°	−1.58	−1.78	3.00	178.4°	−2.71	−2.78
		0.032	2.74	179.4°	−3.28	−3.56	2.81	177.4°	−6.12	−6.30
	PF_3_⋯LB	0.000	3.01	160.5°	−1.89	−2.19	3.07	169.8°	−2.62	−2.62
		0.002	3.00	174.4°	−1.73	−1.96	3.03	171.4°	−2.98	−2.95
		0.004	2.97	175.2°	−1.69	−1.93	2.99	171.9°	−3.40	−3.39
		0.008	2.95	173.1°	−2.10	−2.33	2.90	172.1°	−4.40	−4.40
		0.016	2.83	172.8°	−3.22	−3.48	2.69	172.1°	−7.31	−7.31
		0.032	2.56	171.4°	−6.94	−7.38	2.17	171.6°	−24.36	−24.68
lp-hole	NF_3_⋯LB	0.000	3.53	179.5°	−0.21	−0.32	3.73	179.5°	−0.34	−0.39
		0.002	3.52	179.5°	−0.27	−0.38	3.71	179.6°	−0.42	−0.45
		0.004	3.50	179.6°	−0.33	−0.44	3.70	179.7°	−0.50	−0.55
		0.008	3.44	179.9°	−0.45	−0.56	3.68	179.9°	−0.68	−0.73
		0.016	3.39	180.0°	−0.74	−0.87	3.62	179.9°	−1.12	−1.18
		0.032	3.29	180.0°	−1.49	−1.65	3.54	180.0°	−2.29	−2.40
	PF_3_⋯LB	0.000	3.64	179.2°	−0.09	−0.22	3.78	179.5°	−0.27	−0.34
		0.002	3.61	179.2°	−0.18	−0.32	3.75	180.0°	−0.41	−0.48
		0.004	3.59	179.6°	−0.29	−0.43	3.73	180.0°	−0.57	−0.64
		0.008	3.53	180.0°	−0.51	−0.66	3.67	179.4°	−0.92	−1.00
		0.016	3.42	179.9°	−1.05	−1.22	3.57	179.8°	−1.83	−1.93
		0.032	3.23	179.5°	−2.62	−2.89	3.34	179.9°	−4.76	−4.97

a ∠F–Z⋯LB and Z-centroid⋯LB angles measured within the optimized σ-hole and lp-hole-based complexes, respectively. The centroid was localized between the three coplanar F atoms.

As shown in Fig. 4, negative interaction energies were observed for all considered complexes, indicating the potentiality of the pnicogen-bearing compounds to favorably interact with Lewis bases under the influence of the positively directed EEF and under field-free conditions (*i.e.*, EEF = 0.000 au). For the σ-hole interactions, the Z⋯LB intermolecular distances ranged from 2.17 Å to 3.12 Å, which were less than the sum of the van der Waals (vdW) radii of the two interacting atoms. Additionally, the ∠F–Z⋯LB angles in the σ-hole-based complexes varied from 172.6° to 178.4° and from 171.4° to 175.2° for Z = N and P, respectively, which is highly consistent with previously reported pnicogen⋯LB angles (∠F–Z⋯LB = 170°–180°).^29,76,77^ Exceptionally, the ∠F–P⋯LB angle was found to have a value of 160.5° in PF_3_⋯FH, which formed an undesired interaction.

Based on the results given in Table 2, the interaction energies increased (*i.e.*, became more negative) as the σ-hole size increased in the order NF_3_⋯ < PF_3_⋯LB. For instance, the NF_3_⋯ and PF_3_⋯FH interaction energies under field-free conditions were found to be −0.61 and −1.89 kcal mol^−1^, respectively. Furthermore, it was observed that the interaction energies of the inspected complexes increased as the applied EEF value increased. For example, NF_3_⋯FH exhibited interaction energies of −0.71, −0.81, −1.02, −1.58, and −3.28 kcal mol^−1^ under EEF values of 0.002, 0.004, 0.008, 0.016, and 0.032 au, respectively.

**Table 2:** Topological parameters, including the electron density (*ρ*_b_, au), Laplacian (∇^2^*ρ*_b_, au), and total energy density (*H*_b_, au), at the bond critical points (BCPs) of the optimized ZF_3_⋯FH/NCH complexes (where Z = N and P) under field-free conditions and the positively directed external electric field (EEF)

Complexes		EEF (au)	ZF_3_⋯FH			ZF_3_⋯NCH		
			*ρ*_b_ (au)	∇^2^*ρ*_b_ (au)	*H*_b_ (au)	*ρ*_b_ (au)	∇^2^*ρ*_b_ (au)	*H*_b_ (au)
σ-hole	NF_3_⋯LB	0.000	0.0047	0.0261	0.0016	0.0055	0.0256	0.0016
		0.002	0.0041	0.0231	0.0014	0.0056	0.0264	0.0017
		0.004	0.0050	0.0280	0.0017	0.0058	0.0272	0.0017
		0.008	0.0053	0.0313	0.0019	0.0063	0.0293	0.0018
		0.016	0.0060	0.0354	0.0021	0.0074	0.0341	0.0020
		0.032	0.0080	0.0474	0.0026	0.0119	0.0496	0.0023
	PF_3_⋯LB	0.000	0.0102	0.0433	0.0013	0.0104	0.0346	0.0014
		0.002	0.0084	0.0346	0.0015	0.0115	0.0371	0.0014
		0.004	0.0086	0.0372	0.0017	0.0126	0.0396	0.0013
		0.008	0.0089	0.0379	0.0017	0.0152	0.0452	0.0010
		0.016	0.0113	0.0469	0.0018	0.0237	0.0579	−0.0007
		0.032	0.0202	0.0745	0.0013	0.0690	0.0136	−0.0299
lp-hole	NF_3_⋯LB	0.000	0.0030	0.0144	0.0007	0.0032	0.0127	0.0007
		0.002	0.0031	0.0147	0.0007	0.0034	0.0131	0.0007
		0.004	0.0032	0.0152	0.0007	0.0035	0.0135	0.0007
		0.008	0.0036	0.0168	0.0008	0.0036	0.0139	0.0007
		0.016	0.0039	0.0183	0.0008	0.0040	0.0153	0.0007
		0.032	0.0048	0.0226	0.0010	0.0048	0.0179	0.0008
	PF_3_⋯LB	0.000	0.0032	0.0152	0.0007	0.0038	0.0148	0.0008
		0.002	0.0034	0.0159	0.0007	0.0039	0.0150	0.0008
		0.004	0.0034	0.0161	0.0007	0.0040	0.0157	0.0008
		0.008	0.0037	0.0174	0.0008	0.0045	0.0173	0.0008
		0.016	0.0044	0.0207	0.0009	0.0053	0.0204	0.0009
		0.032	0.0062	0.0291	0.0012	0.0076	0.0292	0.0013

With regard to the lp-hole interactions, the pnicogen⋯LB intermolecular distances were denoted with values in the ranges of 3.3–3.7 Å and 3.2–3.8 Å for Z = N and P, respectively, which exceeded the sum of the vdW radii of the interacting species. Furthermore, the lp-hole⋯LB angles were found to be in the range from 178.2° to 179.9° and from 178.8° to 180.0° for Z = N and P, respectively, indicating the near-linearity of the lp-hole interactions compared to their σ-hole analogs. These observations have been previously reported for the lp-hole interactions in pnicogen-bearing complexes.^30^ From the interaction energy values presented in Table 1, the CCSD(T)/CBS interaction energies of all the lp-hole based complexes under the field-free conditions were −0.32, −0.39, −0.22, and −0.34 kcal mol^−1^ for the NF_3_⋯FH, NF_3_⋯NCH, PF_3_⋯FH, and PF_3_⋯NCH complexes, respectively. This pattern unexpectedly emphasized the further favorability of the nitrogen-bearing complexes to interact *via* the lp-hole with the employed Lewis bases rather than the phosphorus-bearing candidates. Considering the contribution of the positively directed EEF, the potentiality of the phosphorus-bearing molecules to interact with the considered Lewis bases was obviously enhanced, and it became more favorable compared with other candidates, including nitrogen atoms. This observed enhancement can be interpreted as a consequence of the prominent polarization that occurred due to the influence of the utilized EEF. Overall, these results demonstrate the crucial influence of the EEF on the strength of the σ-hole and lp-hole interactions, which is in accord with the MEP maps and *V*_s,max_ values.

### QTAIM analysis

**Fig. 5:**
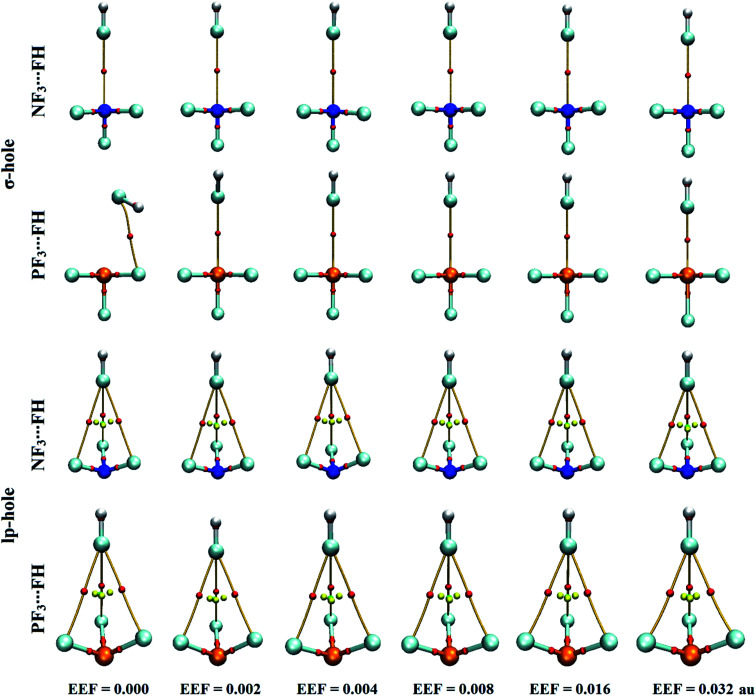
Quantum theory of atoms in molecules (QTAIM) diagrams for σ-hole⋯ and lp-hole⋯FH interactions under the influence of a positively directed external electric field (EEF) and under field-free conditions. The red dots indicate the locations of the bond critical points (BCPs) at the bond paths (BPs).

The quantum theory of atoms in molecules has been successfully adopted to figure out the nature of the interactions.^62^ For the selected pnicogen-bearing complexes, QTAIM analysis was incorporated to reveal the occurrence of the σ-hole and lp-hole interactions by generating bond critical points (BCPs) and bond paths (BPs). Within the context of QTAIM, the nature of the closed-shell interactions was proven and then analyzed through characterization of various BCP features, including the electron density (*ρ*_b_), Laplacian (∇^2^*ρ*_b_), and total energy density (*H*_b_). Fig. 5 shows the BCPs and BPs of the NF_3_⋯ and PF_3_⋯FH complexes optimized under the influence of the positively directed EEF and field-free conditions. For all NF_3_⋯ and PF_3_⋯NCH complexes, the plotted BCPs and BPs are given in Fig. S3.† The extracted *ρ*_b_, ∇^2^*ρ*_b_, and *H*_b_ values of the NF_3_⋯ and PF_3_⋯FH/NCH complexes are collected in Table 2.

As shown in Fig. 5, the occurrence of ZF_3_⋯FH σ-hole interactions was clearly emphasized *via* the existence of the pictorial BP and BCP between the σ-hole of the pnicogen and the fluorine atom, except in the optimized PF_3_⋯FH complex under the field-free conditions. For the lp-hole complexes, three BPs and three BCPs were noted between the three coplanar fluorine atoms of the ZF_3_ molecule and the fluorine atom of the FH Lewis base, indicating the effectual contribution of the latter atoms in the strength of the lp-hole interactions. Additionally, there was no BCP or BP between the examined pnicogen atom and the Lewis base. These findings were found to be highly consistent with the previously recorded emphasis of the minor importance of the BPs in identifying the origin of the considered interactions.^78,79^ Furthermore, the QTAIM of the NF_3_⋯ and PF_3_⋯NCH complexes yielded a picture similar to the corresponding FH analogs; it showed one BP and one BCP for the σ-hole interactions, whereas three were exhibited for the lp-hole candidates (Fig. S3†).

From Table 2, the closed-shell nature was revealed for almost all of the studied pnicogen-bearing complexes based on the relatively low values of *ρ*_b_ and the positive values of ∇^2^*ρ*_b_ and *H*_b_. In line with the energetic results (see Table 1), there was an apparent correlation between the substantial interaction energy of the optimized PF_3_⋯FH complex under the high EEF strength and the negative *H*_b_ values. This observation led us, in turn, to label these complexes as having an eminent covalent nature. A direct correlation was also detected between the *ρ*_b_ values at the BCP and the computed interaction energies under variable EEF strengths. For instance, in the case of the σ-hole interactions, the *ρ*_b_ values in the NF_3_⋯FH complexes were found to be 0.0041, 0.0050, 0.0053, 0.0060, and 0.0080 au with interaction energies of −0.71, −0.81, −1.02, −1.58, and −3.28 kcal mol^−1^ under the influence of EEFs with strengths of 0.002, 0.004, 0.008, 0.016, and 0.032 au, respectively.

### NCI-RDG analysis

**Fig. 6:**
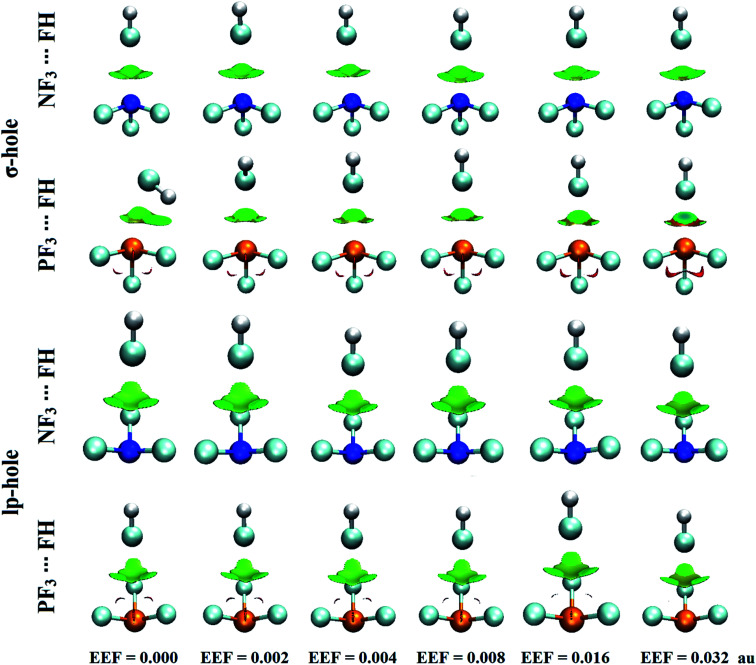
3D NCI plots of the optimized NF_3_⋯ and PF_3_⋯FH complexes under field-free conditions and under the influence of the positively directed external electric field (EEF). The isosurfaces are plotted with a reduced density gradient value of 0.50 au and colored from blue to red according to sign(*λ*_2_)*ρ* ranging from −0.035 au (blue) to 0.020 au (red).

The noncovalent interaction (NCI) index announced by Johnson *et al.* has been deemed a novel descriptor for the nature of the forces beyond various well-established noncovalent interactions based on the reduced density gradient (RDG).^63^ 2D reduced density gradients and 3D color-mapped plots were generated for the complexes under consideration using a color scale of sign(*λ*_2_)*ρ* from −0.035 (blue) to 0.020 (red), where *λ*_2_ is the second eigenvalue of the Hessian matrix and *ρ* is the electron density. Fig. 5 and 6 illustrate the 2D and 3D NCI plots of the optimized ZF_3_⋯FH complexes under the field-free conditions and the influence of the positively directed external electric field (EEF). For the optimized ZF_3_⋯NCH complexes, the 2D and 3D NCI plots are displayed in Fig. S5 and S6.†

It can be observed first from the 2D NCI plots presented in Fig. S4 and S5† that all the spikes are located at negative values of sign(*λ*_2_)*ρ*, confirming the attractive interactions between the two interacting species.

According to Fig. 6, green regions were denoted between the two interacting monomers, confirming the occurrence of weak σ-hole and lp-hole interactions in the studied complexes. Notably, a direct correlation was found between the positively directed EEF strength and the size of the green isosurfaces (*i.e.*, the green isosurface size was increased by increasing the value of the positively directed EEF). For the NF_3_⋯ and PF_3_⋯NCH complexes, as apparently noted in Fig. S6,† the largest size of the green isosurfaces occurred within the optimized complexes under the influence of the positively directed EEF with a value of 0.032 au. Moreover, a larger expanded area of the green isosurfaces was observed for the σ-hole interactions compared to the lp-hole analogs, indicating the favorability of σ-hole interactions over lp-hole ones.

### Point-of-charge (PoC) calculations

For some years, the point-of-charge (PoC) approach has been recommended as an efficient tool to predict the potentiality of group III–VII elements to engage in purely electrostatic interactions.^30,80–84^ With the execution of the PoC calculations, negative PoC was used to imitate the effect of the Lewis base on the examined pnicogen-bearing systems. The NF_3_⋯ and PF_3_⋯PoC systems were scanned in the presence of −0.25, −0.50, −0.75, and −1.00 au PoCs at σ-hole/lp-hole⋯PoC distances ranging from 2.5 to 6.0 Å with a step size of 0.1 Å (see the computational methods section for details). Molecular stabilization energy curves for the optimized monomers under the influence of the positively directed external electric field (EEF) and the field-free conditions were generated and are illustrated in Fig. S7.†Table 3 compiles the values of the molecular stabilization energies computed at a σ-hole/lp-hole⋯PoC distance of 2.5 Å under the field-free conditions and under the influence of the positively directed EEF.

**Table 3:** Molecular stabilization energies for the σ-hole⋯ and lp-hole⋯PoC interactions in the ZF_3_⋯PoC systems (where Z = N and P) calculated at a Z⋯PoC distance of 2.5 Å under the field-free conditions and the influence of the positively directed external electric fields (EEFs) with values ranging from 0.002 to 0.032 au in the presence of PoC values of −0.25, −0.50, −0.75, and −1.00 au

Complexes		EEF (au)	Molecular stabilization energies (*E*_stabilization_, kcal mol^−1^)			
			−0.25	−0.50	−0.75	−1.00
σ-hole	NF_3_⋯LB	0.000	−1.52	−3.99	−7.35	−11.57
		0.002	−1.66	−4.26	−7.75	−12.10
		0.004	−1.78	−4.49	−8.10	−12.56
		0.008	−2.05	−5.03	−8.89	−13.62
		0.016	−2.56	−6.04	−10.40	−15.61
		0.032	−3.57	−8.06	−13.43	−19.66
	PF_3_⋯LB	0.000	−4.52	−10.80	−18.66	−27.96
		0.002	−4.73	−11.21	−19.25	−28.73
		0.004	−4.94	−11.61	−19.83	−29.49
		0.008	−5.36	−12.42	−21.00	−31.01
		0.016	−6.19	−14.01	−23.34	−34.07
		0.032	−7.84	−17.26	−28.19	−40.57
lp-hole	NF_3_⋯LB	0.000	−0.28	−1.37	−3.24	−5.85
		0.002	−0.43	−1.67	−3.68	−6.43
		0.004	−0.59	−1.97	−4.12	−7.02
		0.008	−0.89	−2.57	−5.00	−8.18
		0.016	−1.49	−3.75	−6.76	−10.50
		0.032	−2.68	−6.11	−10.28	−15.17
	PF_3_⋯LB	0.000	0.13	−0.73	−2.56	−5.33
		0.002	−0.13	−1.26	−3.34	−6.35
		0.004	−0.40	−1.78	−4.11	−7.37
		0.008	−0.93	−2.82	−5.65	−9.40
		0.016	−1.98	−4.90	−8.73	−13.47
		0.032	−4.13	−9.17	−15.11	−21.95

As shown in Table 3, the results indicate the sizeable contribution of the PoC negativity (*i.e.*, Lewis basicity) to the strengths of the σ-hole and lp-hole interactions of the pnicogen-bearing molecules. Evidently, the molecular stabilization energy increased as the negativity of the incorporated PoC increased. For example, the σ-hole⋯PoC molecular stabilization energies of −4.52, −10.80, −18.66, and −27.96 kcal mol^−1^ were observed for PF_3_ molecule under field-free conditions by incorporating −0.25, −0.50, −0.75, and −1.00 au PoCs, respectively.

Additionally, the molecular stabilization energy decreased (*i.e.*, became less negative) as the σ-hole/lp-hole⋯PoC distance increased under the field-free conditions and the positively directed EEF influence (Fig. S7†). According to the results, the σ-hole interactions showed more favorable negative molecular stabilization energies compared with their lp-hole analogs. For the PF_3_⋯PoC system, as an example, the molecular stabilization and destabilization energies in the presence of −0.25 au PoC under field-free conditions were −4.52 and 0.13 kcal mol^−1^ for the σ-hole and lp-hole interactions, respectively.

Moreover, a direct correlation was observed between the σ-hole magnitude of the pnicogen-bearing molecule and the molecular stabilization energy. As an example, NF_3_ and PF_3_ exhibited −1.66 and −4.73 kcal mol^−1^ in the presence of a −0.25 au PoC under the influence of a +0.002 au EEF (Table 3).

On the other hand, the molecular stabilization energies of the lp-hole electrostatic interactions showed an inverse correlation with the lp-hole magnitude (*i.e.*, the atomic size of the pnicogens) under the field-free conditions and the influence of an entirely weak positively directed EEF. For instance, under field-free conditions, the molecular stabilization energies of the lp-hole interactions in the presence of a −0.25 au PoC were recorded with values of −0.28 and 0.13 kcal mol^−1^ for NF_3_⋯ and PF_3_⋯PoC, respectively. It is also worth noting that the versatility of PF_3_ molecule to interact *via* the lp-hole was enhanced, with more favorable molecular stabilization energies compared to the NF_3_ molecule, only by depositing EEFs with high strength in the positive direction along the *z*-axis. In accord with the interaction energy pattern, the molecular stabilization energies for the lp-hole interactions in the studied pnicogen-bearing systems were recognized to have an inverse correlation with the *V*_s,max_ values under the field-free conditions and an entirely weak EEF strength.

Turning to the EEF results, the positively directed EEF gave rise to intriguing potency of the discussed systems to participate in σ-hole and lp-hole interactions. With numerical evidence, taking the σ-hole interactions of the PF_3_⋯PoC system as an example, in the presence of −0.25 au PoC, the molecular stabilization energies were −4.52, −4.73, −4.94, −5.36, −6.19, and −7.84 kcal mol^−1^ under the field-free conditions and 0.002, 0.004, 0.008, 0.016, and 0.032 au EEF values, respectively. These informative findings emphasized the prominent role of the EEF in controlling the solid energetic features of the noncovalent interactions, which is in line with the interaction energy trend of the examined complexes (see the Energetic study section).

### Lewis basicity effect

**Fig. 7:**
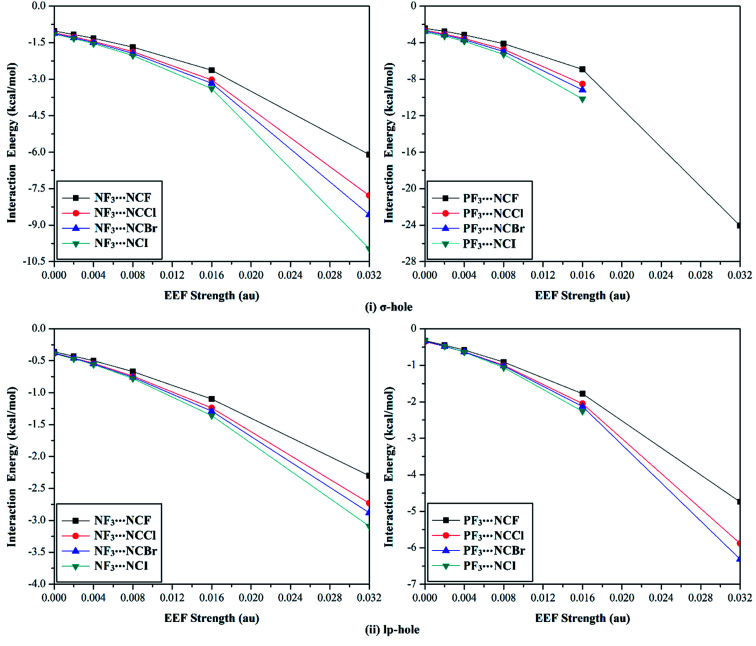
Interaction energy curves of the NF_3_⋯ and PF_3_⋯NCX (where X = F, Cl, Br, and I) complexes calculated under the field-free conditions and the influence of the positively directed external electric field (EEF) for (i) σ-hole⋯ and (ii) lp-hole⋯NCX interactions.

To further understand the σ-hole and lp-hole interactions, the effects of Lewis basicity on the strength of the pnicogen-bearing complexes was herein addressed *via* using halogen substituents in the NCX Lewis base (where X = F, Cl, Br, and I). For the NF_3_⋯ and PF_3_⋯NCX complexes, the geometrical optimization was first performed under the positively directed EEF influence and the field-free conditions at MP2/aug-cc-pVTZ(PP) level of theory (see the computational methods section for details). The corresponding interaction energies were then computed for the optimized complexes at the same level of geometrical optimization (Table 4). Fig. 7 provides the intercorrelation between the interaction energy of the NF_3_⋯ and PF_3_⋯NCX complexes and the employed positively directed EEF strength. Missing data in Fig. 7 and Table 4 resulted as a consequence of the covalent bond formation between the ZF_3_ and NCX species.

**Table 4:** Interaction energies (*E*) calculated (in kcal mol^−1^) at the MP2/aug-cc-pVTZ(PP) level of theory for the optimized complexes under the influence of the positively directed external electric field (EEF) and the field-free conditions for σ-hole⋯ and lp-hole⋯NCX (where X = F, Cl, Br, and I) interactions

Complexes		EEF (au)	ZF_3_⋯NCF		ZF_3_⋯NCCl		ZF_3_⋯NCBr		ZF_3_⋯NCI	
			Distance (Å)	*E* (kcal mol^−1^)	Distance (Å)	*E* (kcal mol^−1^)	Distance (Å)	*E* (kcal mol^−1^)	Distance (Å)	*E* (kcal mol^−1^)
σ-hole	NF_3_⋯LB	0.000	3.11	−1.02	3.10	−1.10	3.08	−1.12	3.08	−1.15
		0.002	3.09	−1.16	3.08	−1.26	3.08	−1.30	3.07	−1.33
		0.004	3.07	−1.32	3.07	−1.45	3.06	−1.49	3.05	−1.54
		0.008	3.05	−1.68	3.03	−1.87	3.02	−1.94	3.02	−2.03
		0.016	2.98	−2.63	2.96	−3.03	2.95	−3.17	2.94	−3.39
		0.032	2.80	−6.10	2.73	−7.78	2.69	−8.57	2.63	−9.79
	PF_3_⋯LB	0.000	3.09	−2.43	3.06	−2.64	3.05	−2.74	3.03	−2.84
		0.002	3.04	−2.75	3.02	−3.05	2.99	−3.15	2.97	−3.28
		0.004	3.00	−3.15	2.97	−3.54	2.95	−3.67	2.93	−3.85
		0.008	2.92	−4.11	2.86	−4.73	2.84	−4.97	2.81	−5.29
		0.016	2.71	−6.92	2.61	−8.53	2.58	−9.18	2.53	−10.17
		0.032	2.17	−24.05	—a	—a	—a	—a	—a	—a
lp-hole	NF_3_⋯LB	0.000	3.70	−0.36	3.69	−0.39	3.67	−0.39	3.66	−0.39
		0.002	3.68	−0.43	3.67	−0.46	3.66	−0.46	3.65	−0.47
		0.004	3.67	−0.50	3.66	−0.54	3.64	−0.55	3.64	−0.56
		0.008	3.64	−0.67	3.64	−0.74	3.63	−0.76	3.62	−0.78
		0.016	3.59	−1.10	3.58	−1.24	3.58	−1.29	3.57	−1.36
		0.032	3.51	−2.30	3.50	−2.73	3.49	−2.88	3.49	−3.09
	PF_3_⋯LB	0.000	3.76	−0.32	3.75	−0.35	3.73	−0.34	3.73	−0.33
		0.002	3.73	−0.45	3.72	−0.48	3.71	−0.48	3.70	−0.47
		0.004	3.72	−0.58	3.69	−0.63	3.68	−0.63	3.67	−0.64
		0.008	3.65	−0.91	3.64	−1.00	3.63	−1.02	3.62	−1.06
		0.016	3.55	−1.78	3.53	−2.05	3.52	−2.13	3.51	−2.26
		0.032	3.33	−4.74	3.29	−5.88	3.27	−6.31	—a	—a

a The optimum structure cannot be achieved due to covalent bond formation between the interacting species.

According to the data presented in Fig. 7, a direct correlation was noted between the interaction energies and the values of the utilized EEF (*i.e.*, the interaction energy increased as the EEF value increased). As an illustration, favorable σ-hole interactions were found for the studied NF_3_⋯NCF complexes, with interaction energy values of −1.02, −1.16, −1.32, −1.68, −2.63, and −6.10 kcal mol^−1^ under the 0.000, 0.002, 0.004, 0.008, 0.016, and 0.032 au EEFs, respectively. The same pattern was denoted for the lp-hole⋯NCX interactions with low interaction energy values compared with the σ-hole analogs. As a point of comparison, the interaction energies of the optimized NF_3_⋯NCF complexes under the field-free conditions were obtained, with values of −1.02 and −0.36 kcal mol^−1^ for σ-hole and lp-hole interactions, respectively. These results illustrate the greater favorability of the lp-hole interactions than their σ-hole counterparts, as mentioned in the EP analysis and energetic study sections.

Looking at Table 4, it is apparent that all the incorporated Lewis bases were observed to have impressive potentiality to participate in the σ-hole and lp-hole interactions of pnicogens, and considerable interaction energies were recorded. For σ-hole⋯NCX interactions, favorable interaction energies with significant values were noted, and these values increased with increasing X atomic size (*i.e.*, in the order ZF_3_⋯NCF < ⋯NCCl < ⋯NCBr < ⋯NCI). For instance, interaction energies were found with values of −1.02, −1.10, −1.12, and −1.15 kcal mol^−1^ for the field-free optimized NF_3_⋯NCF, ⋯NCCl, ⋯NCBr, and ⋯NCI complexes, respectively.

## Conclusion

The current study provides a fully characterized picture of the σ-hole and lp-hole interactions in pnicogen-bearing complexes, for the first time, under external electric field (EEF) and field-free conditions. σ-holes and lp-holes obviously occurred in all the selected pnicogen-bearing molecules, with variable sizes depending on the atomic size of the examined pnicogen and the directionality and strength of the employed EEF. Remarkably, an unanticipated effect was found for the strong negatively directed EEF (*i.e.*, large EEF value) on the lp-hole size, demonstrating the larger lp-hole sizes for nitrogen-bearing monomers than for phosphorus-bearing ones. Under the field-free conditions and the influence of a positively directed EEF, the MP2 results disclosed the further favorability of the σ-hole interactions compared to their lp-hole analogs, with substantial negative interaction energies. The PF_3_⋯LB complexes exhibited more impressive interaction energies than the nitrogen-bearing complexes, in particular, with increasing positively directed EEF value for the lp-hole interactions. Unexpectedly, the NF_3_-bearing complexes were evidently observed to have the most significant interaction energies for the lp-hole interactions under field-free conditions and weak positively directed EEF strength (*i.e.*, small EEF value). Point-of-charge (PoC) calculations confirmed the preferential versatility of the examined pnicogens to interact *via* σ-holes more than lp-holes, with considerable negative molecular stabilization energies. These outstanding findings confirm the eminent role of a directed EEF in tuning the strength of group V interactions.

## Conflicts of interest

There are no conflicts to declare.

## Supplementary Material

RA-011-D0RA09765A-s001
