# Supplementary material for: External electric field effects on the σ-hole and lone-pair hole interactions of group V elements: a comparative investigation
Source: RSC Adv. 2021 Jan 19;11(7):4022–34. doi: 10.1039/d0ra09765a (PMC8694126; doi:10.1039/d0ra09765a)
Supplement: RA-011-D0RA09765A-s001 [file RA-011-D0RA09765A-s001.pdf]

Electronic Supplementary Material (ESI) for RSC Advances.
This journal is © The Royal Society of Chemistry 2021

External electric field effect on σ-hole and lone-pair hole interactions of Group V elements: A comparative investigation

Mahmoud A. A. Ibrahim,[1,*] Sherif M. A. Saad,[1] Jabir H. Al-Fahemi,[2] Gamal A. H. Mekhemer,[1] Saleh A. Ahmed,[2,3] Ahmed M. Shawky[4,5] and Nayra A. M. Moussa[1]

[1] Computational Chemistry Laboratory, Chemistry Department, Faculty of Science, Minia University, Minia 61519, Egypt

[2]Chemistry Department, Faculty of Applied Sciences, Umm Al-Qura University, Makkah 21955, Saudi Arabia

[3]Chemistry Department, Faculty of Science, Assiut University, Assiut 71516, Egypt

[4]Science and Technology Unit (STU), Umm Al-Qura University, Makkah 21955, Saudi Arabia

[5]Central Laboratory for Micro-analysis, Minia University, Minia 61519, Egypt

* Corresponding author: Mahmoud A. A. Ibrahim; m.ibrahim@compchem.net

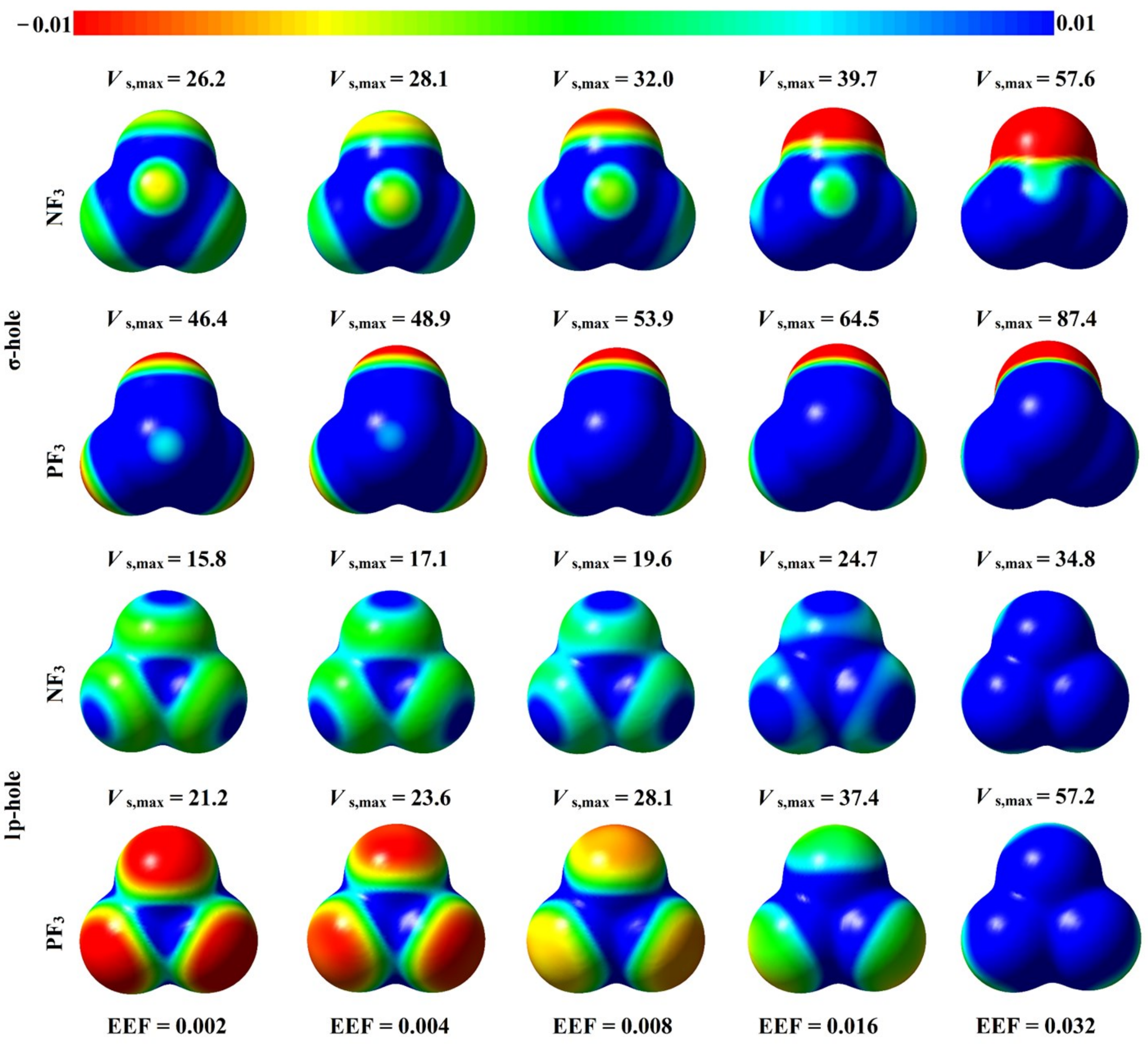

Figure S1. Molecular electrostatic potential (MEP) maps plotted onto 0.002 au electron density contours under the influence of positively-directed external electric field (EEF) (values in au). The electrostatic potential varies from −0.01 (red) to +0.01 (blue) au. The calculated maximum positive electrostatic potentials ($V_{s,max}$) at σ-hole and lp-hole under the directed EEF in NF_3 and PF_3 molecules are given in kcal/mol.

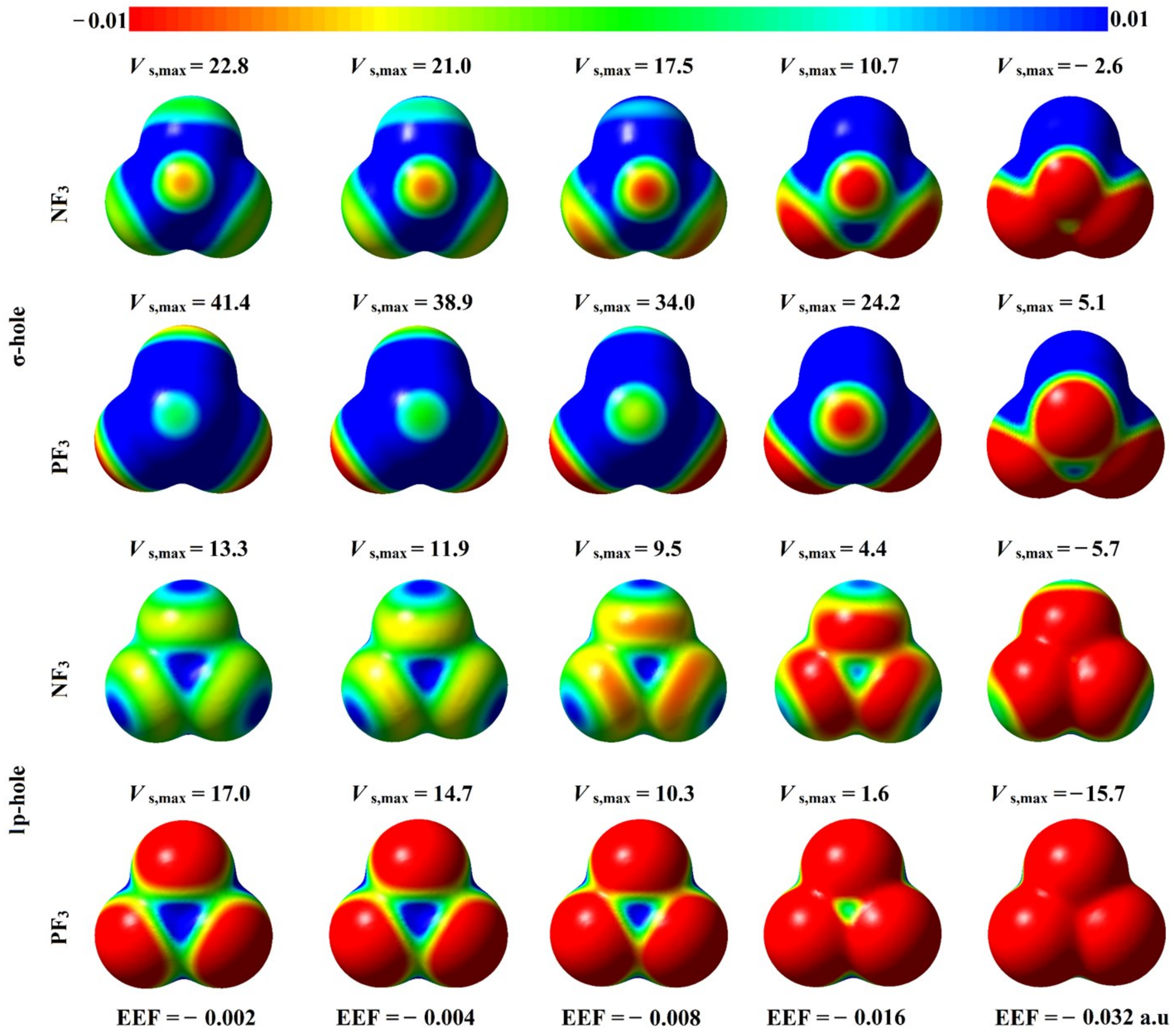

Figure S2. Molecular electrostatic potential (MEP) maps plotted onto 0.002 au electron density contours under the influence of negatively-directed external electric field (EEF) (values in au). The electrostatic potential varies from −0.01 (red) to +0.01 (blue) au. The calculated maximum positive electrostatic potentials ($V_{s,max}$) at σ-hole and lp-hole under the directed EEF in NF_3 and PF_3 molecules are given in kcal/mol.

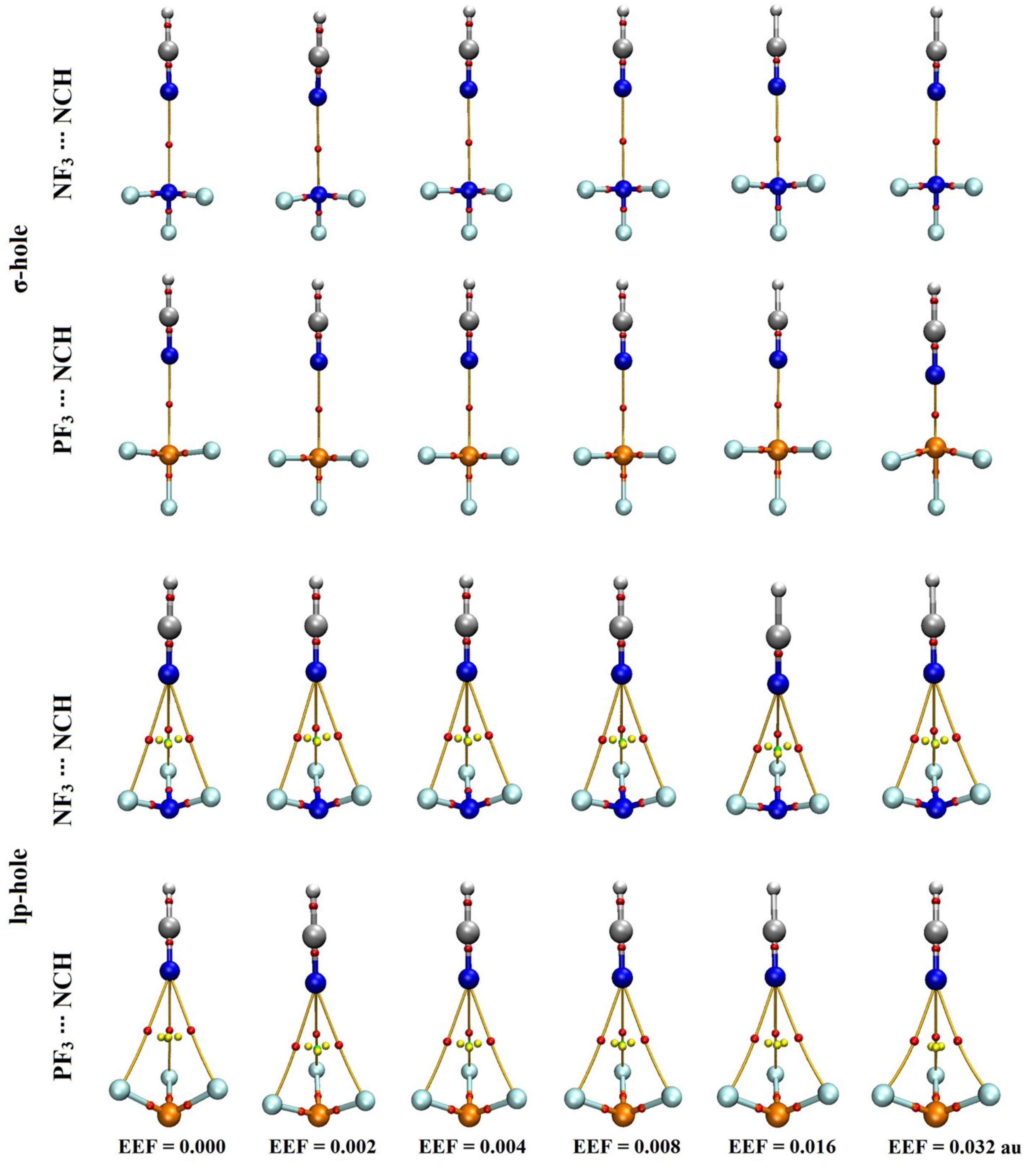

Figure S3. Quantum theory of atoms in molecules (QTAIM) diagrams for σ-hole··· and lp-hole···NCH interactions under the field-free conditions and the influence of the positively-directed external electric field (EEF). Red dots indicate the locations of bond critical points (BCPs) at bond paths (BPs).

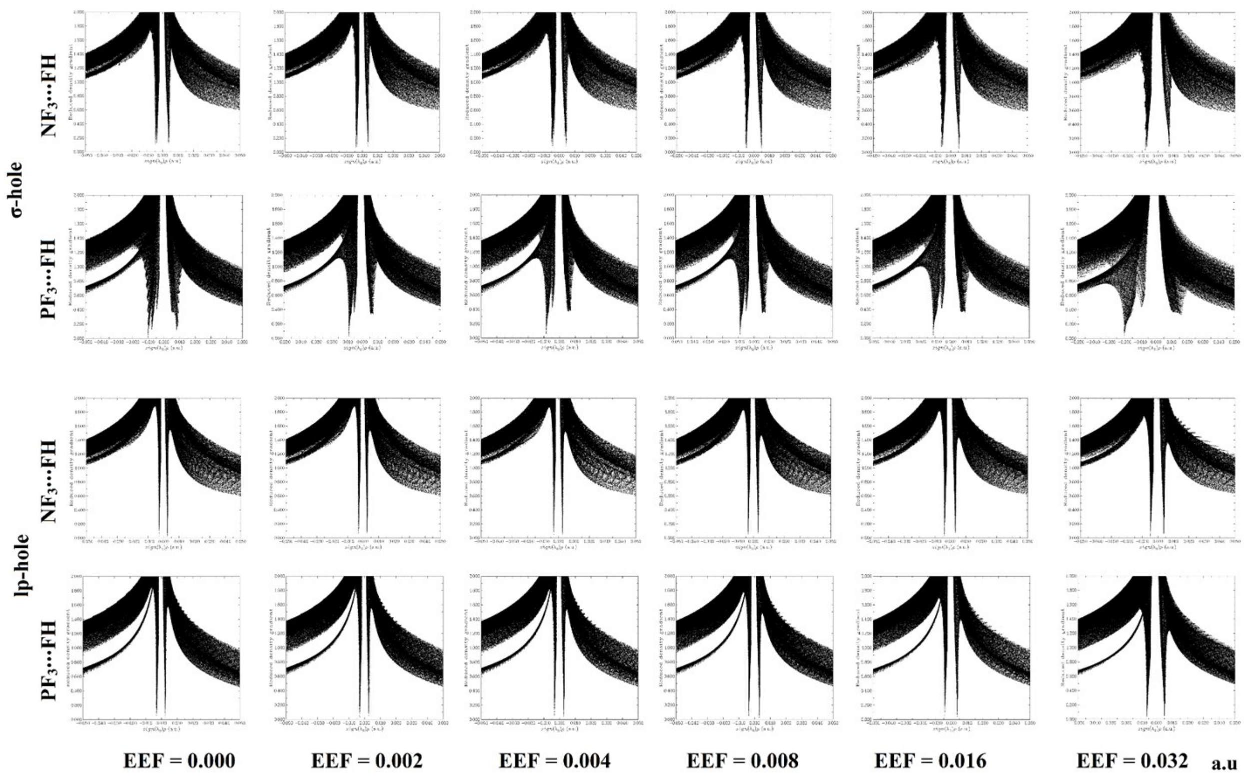

Figure S4. 2D NCI diagrams of the RDG versus the electron density multiplied by the sign of the second Hessian eigenvalue for the optimized NF_3··· and PF_3···FH complexes under the field-free conditions and the influence of the positively-directed external electric field (EEF).

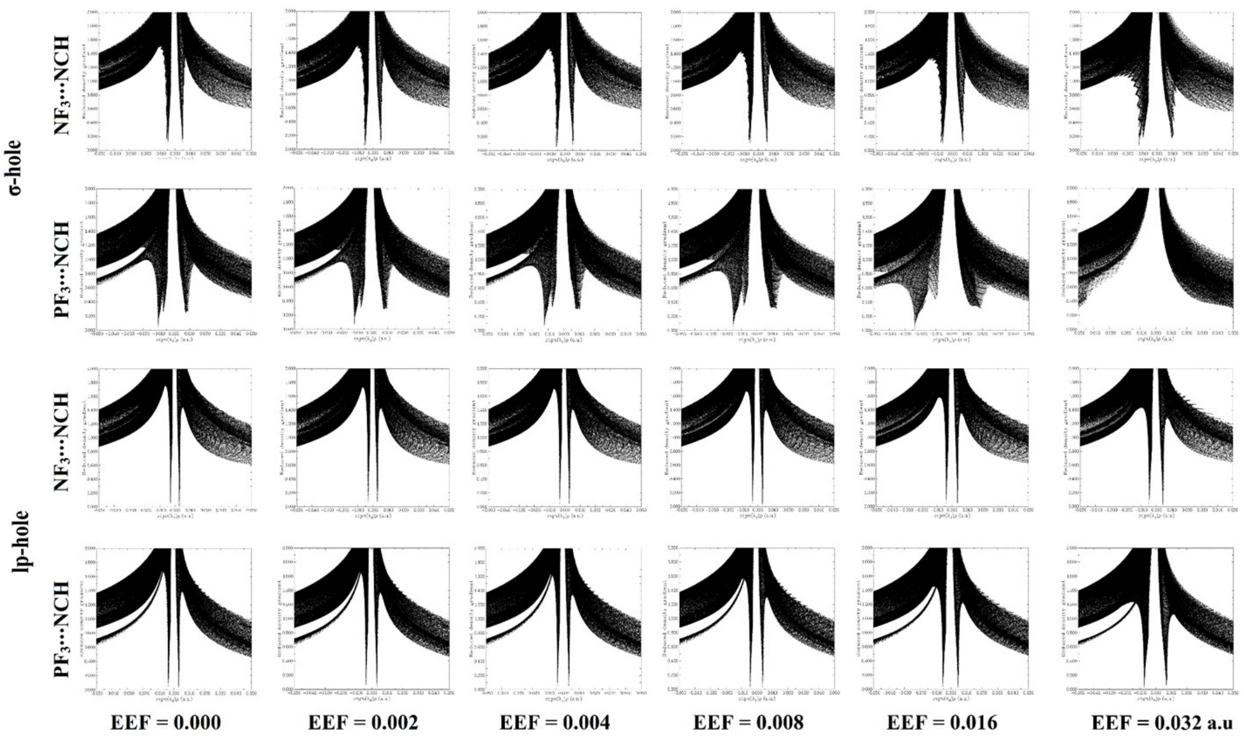

Figure S5. 2D NCI diagrams of the RDG versus the electron density multiplied by the sign of the second Hessian eigenvalue for the optimized NF_3··· and PF_3···NCH complexes under the field-free conditions and the influence of the positively-directed external electric field (EEF).

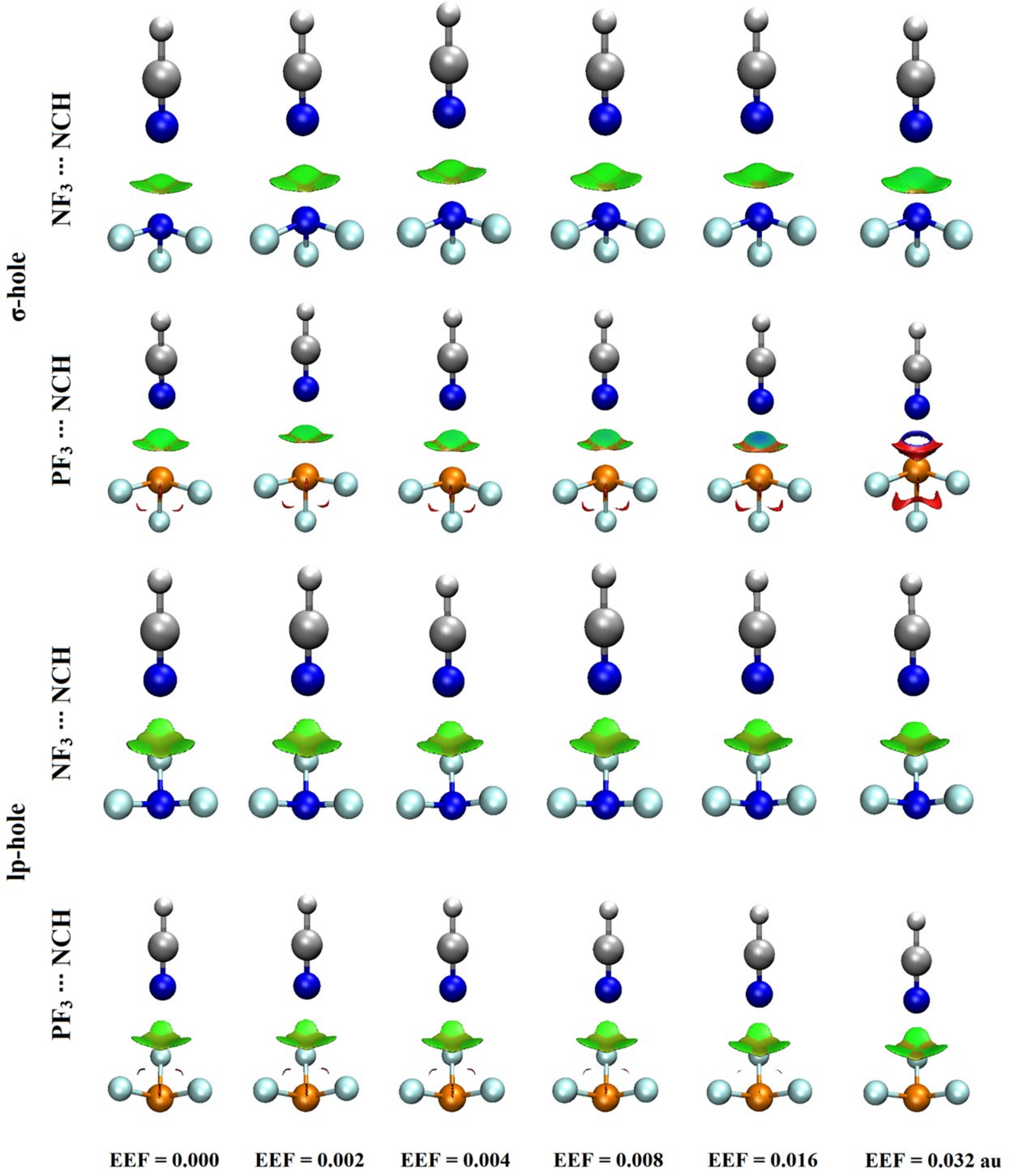

Figure S6. 3D NCI plots of for the optimized NF_3··· and PF_3···NCH complexes under the field-free conditions and the influence of the positively-directed external electric field (EEF). The isosurfaces plotted with a reduced density gradient value of 0.50 au and colored from blue to red according to sign(λ_2)ρ ranging from −0.035 (blue) to 0.020 (red) au.

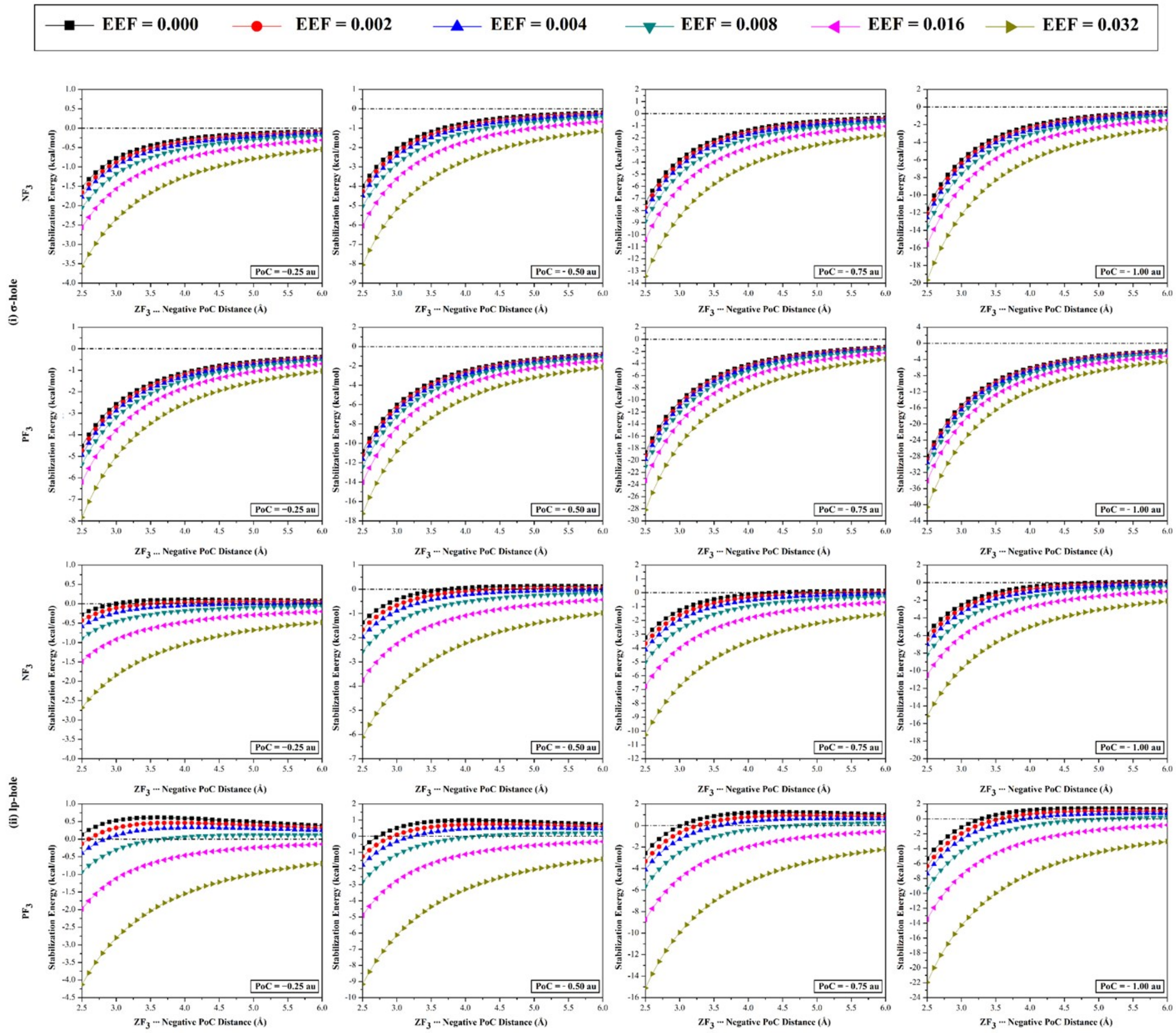

Figure S7. Molecular stabilization energy curves for (i) σ-hole··· and (ii) lp-hole···PoC interactions in the ZF_3···PoC systems (where Z = N and P) calculated at Z···PoC distance range of 2.5–6.0 Å under the field free condition and the influence of the positively-directed external electric field (EEF) with values ranging from 0.002 to 0.032 au in the presence of PoC values of –0.25, –0.50, –0.75, and –1.00 au.
